# Impact of imposed social isolation and use of face masks on asthma course and mental health in pediatric and adult patients with recurrent wheeze and asthma

**DOI:** 10.1186/s13223-021-00592-9

**Published:** 2021-09-16

**Authors:** Nicole Maison, Heidrun Herbrüggen, Bianca Schaub, Christina Schauberger, Svenja Foth, Ruth Grychtol, Mustafa Abdo, Henrik Watz, Wilfried Nikolaizik, Klaus F. Rabe, Matthias V. Kopp, Gesine Hansen, Erika von Mutius, Thomas Bahmer, Jimmy Omony, the ALLIANCE study group, Oliver Fuchs, Barbara Roesler, Nils Welchering, Naschla Kohistani-Greif, Johanna Kurz, Katja Landgraf-Rauf, Kristina Laubhahn, Nicole Maison, Bianca Schaub, Markus Ege, Erika von Mutius, Sabina Illi, Jimmy Omony, Alexander Hose, Esther Zeitlmann, Mira Berbig, Carola Marzi, Christina Schauberger, Isabell Ricklefs, Gesa Diekmann, Lena Liboschik, Gesche Voigt, Laila Sultansei, Markus Weckmann, Matthias V. Kopp, Gyde Nissen, Inke R. König, Dominik Thiele, Thomas Bahmer, Anne-Marie Kirsten, Frauke Pedersen, Henrik Watz, Benjamin Waschki, Klaus F. Rabe, Christian Herzmann, Lukas Hundack, Annika Opitz, Karoline I. Gaede, Xenia Bovermann, Alena Steinmetz, Vera Veith, Folke Brinkmann, Anna-Maria Dittrich, Christine Happle, Ruth Grychtol, Aydin Malik, Nicolaus Schwerk, Christian Dopfer, Mareike Price, Gesine Hansen, Adan Chari Jirmo, Anika Habener, David S. DeLuca, Wilfried Nikolaizik, Michael Zemlin, Svenja Foth, Annemiek Leson, Anna Werlein, Nele Maier, Chrysanthi Skevaki, Harald Renz, Tom Schildberg, Ernst Rietschel, Silke van Koningsbruggen-Rietschel

**Affiliations:** 1 Department of Paediatric Allergology, Dr Von Hauner Children’s Hospital, Ludwig Maximilians University, Munich, Germany; 2 Comprehensive Pneumology Center, Munich (CPC-M), Munich, Germany; 3 German Center for Lung Research (DZL), Munich, Germany; 4 Institut Für Asthma- Und Allergieprävention (IAP), Helmholtz Zentrum Munich, Deutsches Forschungszentrum Für Gesundheit Und Umwelt (GmbH), Munich, Germany; 5 LungenClinic Grosshansdorf GmbH, Grosshansdorf, Germany; 6 Airway Research Center North (ARCN), Grosshansdorf, Germany; 7 University Children’s Hospital Marburg, University of Marburg, Marburg, Germany; 8 University of Giessen Marburg Lung Center (UGMLC), Giessen, Germany; 9 Department of Paediatric Pneumology, Allergology and Neonatology, Hannover Medical School, Hannover, Germany; 10 Biomedical Research in Endstage and Obstructive Lung Disease Hannover (BREATH), Hannover, Germany; 11 Pulmonary Research Institute at LungenClinic Grosshansdorf, Grosshansdorf, Germany; 12 Institute for Medical Biometry and Statistics, University Luebeck, University Medical Centre Schleswig-Holstein, Campus, Luebeck, Germany; 13 Internal Medicine Department I, University Hospital Schleswig-Holstein, Campus Kiel, Kiel, Germany

**Keywords:** COVID-19, Asthma, Side effects, Psychological health, Health care utilization, Facemasks

## Abstract

**Background:**

There is currently a dramatic increase in the number of COVID-19 cases worldwide, and further drastic restrictions in our daily life will be necessary to contain this pandemic. The implications of restrictive measures like social-distancing and mouth-nose protection on patients with chronic respiratory diseases have hardly been investigated.

**Methods:**

Our survey, was conducted within the All Age Asthma Cohort (ALLIANCE), a multicenter longitudinal observational study. We assessed the effects of COVID-19 imposed social isolation and use of facial masks, on asthma course and mental health in patients with asthma and wheezing.

**Results:**

We observed a high rate of problems associated with using facemasks and a significant reduction in the use of routine medical care. In addition to unsettling impacts, such as an increase in depression symptoms in adults, an astonishing and pleasing effect was striking: preschool children experienced an improvement in disease condition during the lockdown. This improvement can be attributed to a significant reduction in exposure to viral infections.

**Conclusion:**

Long-term observation of this side effect may help improve our understanding of the influence of viral infections on asthma in early childhood.

## To the Editor

### Background

Since the start of the COVID-19 pandemic, nearly 135.5 million people have been infected worldwide, with almost 2.93 million deaths recorded—as of mid-April 2021 [1]. The major implications due to social-restrictions, thoroughly applied strategies to decrease transmission of SARS-Cov-2 and general adaptations in healthcare delivery have become increasingly evident.

During the shutdown, world-wide pediatric emergency departments experienced a substantial drop in the number of patient visits [2]. A delayed seeking of care in critical medical conditions was frequently reported in adult patients [3]. Furthermore, symptoms of depression have been reported to be at least 3-fold higher in adults during COVID-19 compared to before the pandemic [4]. As part of the strategy to reduce transmission of SARS-CoV-2, on April 27th 2020, several regions in Germany introduced a provision of mouth-nose protection in public places. Facemasks differ in their effectiveness, depending on the grade of droplet deposition and are one of the most effective intervention strategies against COVID-19 [5]. Studies suggest that adult patients with acute or chronic lung diseases and pre-existing breathing difficulties experience shortness of breath when using surgical facemasks [6].

### Methods

There is lack of data related to the indirect impact of COVID-19, due to social distancing and use of facial masks in children with asthma, although asthma is the most frequent chronic airway disease in children. Therefore, we investigated the impact of measures undertaken to prevent the further spread of SARS-CoV-2 in the public on the general health-related quality of life of children, adolescents and adults in a pre-existing longitudinal cohort of patients with wheeze or asthma. We assessed health-care utilization, tolerance of wearing facemasks, psychological health and disease-related symptoms.

We conducted a questionnaire-based study within the clinical All Age Asthma Cohort (ALLIANCE)—a prospective, multicenter cohort of pre-school children with recurrent wheeze and children, adolescents and adults with asthma from several sites of the German Center of Lung Research (DZL) (Munich, Hannover, Grosshansdorf, Marburg). The inclusion criteria were at least two episodes of wheeze (children < 6 years) or an asthma diagnosis according to GINA guidelines (children ≥ 6 years and adults) [7]. The prevalence and intensity of depression symptoms were determined using the Patient Health Questionnaire-9 in children older than 12 years and adults. The depression categories are: none (score 0–4), mild (score 5–9), moderate (score 10–14), moderately severe (score 15–19), and severe (score ≥ 20). Questionnaires were answered by the families at home or during one of the regular hospital study visits. The local ethics committees approved the study and all participants gave their written informed consent.

### Results

**Fig. 1:**
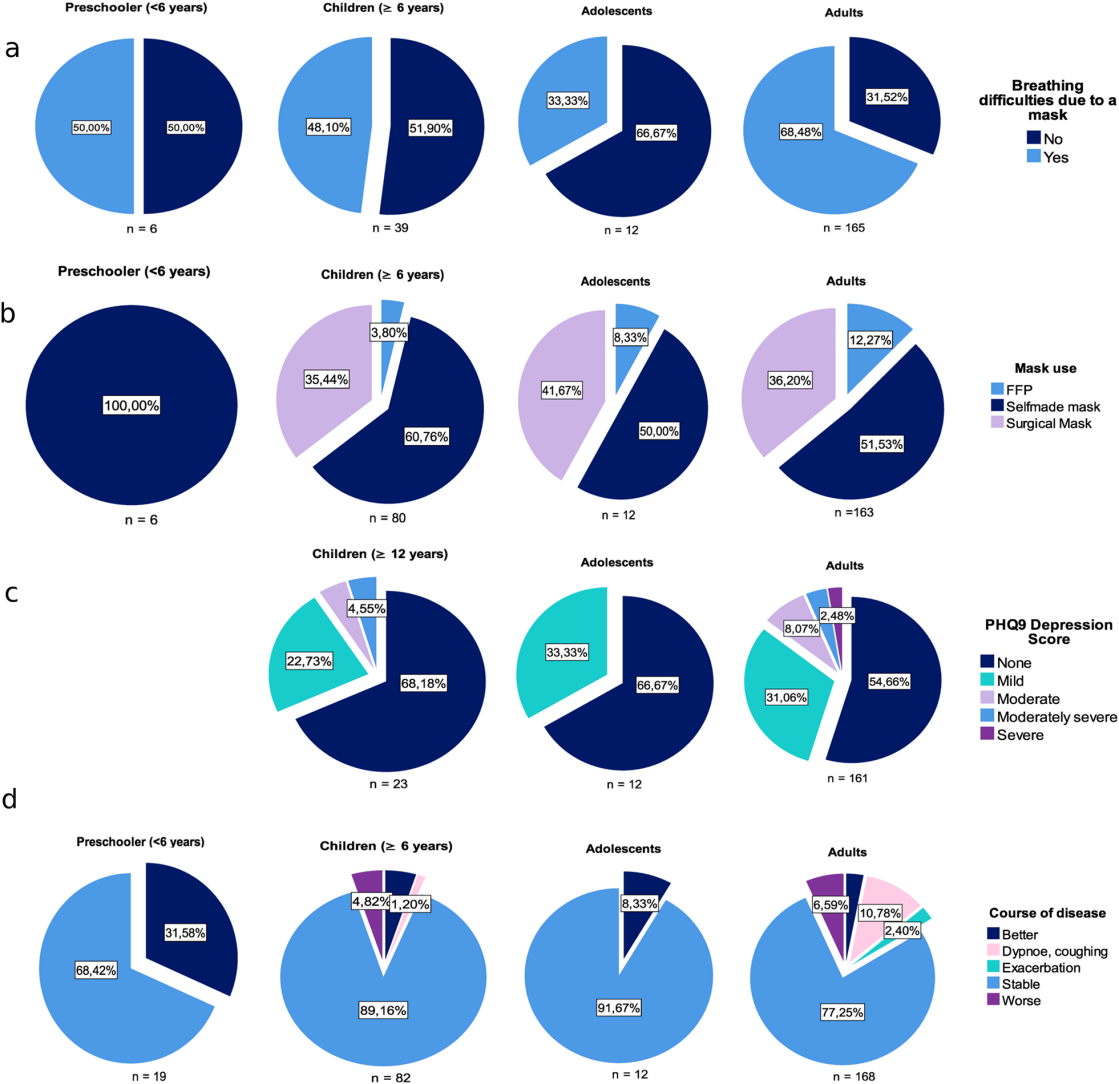
Impact of COVID-19 related measures on children, adolescents and adults

Between June–August 2020 a total of n = 282 subjects of the ALLIANCE cohort participated in the study. Nineteen preschoolers (of whom n = 15, 78.9% were male), n = 82 school-age children (n = 62, 75.61% male), n = 12 adolescents (n = 6, 50% male) and 168 adults (n = 73, 43.5% male) answered the questionnaire. Overall, two adolescents (16.7%), one preschooler (5.3%), one child (1.2%) and five adults (3.0%) had contact with an index patient infected with SARS-CoV-2. However, there were no infected patients in this cohort at that time. Forty-five percent of the subjects reported missing their regular doctor appointment in the first half of the year 2020 due to the pandemic. The appointments were cancelled in equal parts by the patient and by their doctor or clinic. Only 5.5–8.5% of the appointments were substituted by a telephone visit—a proportion that did not differ across age-groups. At the time point of this assessment only 3. 8% (in children > 6 years) to 12.3% (in adults) wore FFP2 masks. The percentage of surgical masks (35.4–41.7%) and self-made masks (100% < 6 years old, but 50–60% in older children and adults) were similar between age-groups. All age-groups complained of mask-related breathing difficulties, disturbing smell, heat, sweating and skin problems (data not shown)—regardless of the type of mask used (Fig. 1a and b).

The PHQ-9 depression score revealed mild and moderate-to-severe symptoms among 31% and 14% of adult asthma patients, respectively. Before the pandemic, 8.9% of included patients were diagnosed with depression, where females were more affected 13.7% (13/95) than males 2.9% (2/70); (Chi-square = 4.48, p = 0.034). A higher depression score (PHQ-9, dichotomized: low versus high) was related to a preexisting diagnosis of depression (${\chi }_{1}^{2}$, p < 0.001). However, 14% of the subjects who reported symptoms of depression during the pandemic had no history of depression. In contrast, only 8% of the children (> 12–17 years) and none of the adolescents (17–23 years) showed moderate-to-severe depression symptoms (Fig. 1c). Twenty percent of adults reported worsening of asthma. Both depression and worsening of illness were unrelated to health-care access. Using dichotomized depression scores (none versus mild-to-severe), and asthma conditions (uncontrolled versus controlled), we observed a strong positive association between depression score and disease condition (${\chi }_{1}^{2}$, p = 0.002).

Adolescent patients reported mostly stable (92%) or improved asthma (8%). Interestingly, almost a third (32%) of parents of the preschool children reported improved course of disease since the beginning of the pandemic (Fig. 1d). No infection with SARS-CoV-2 was reported among asthmatics. The prevalence of asthma among SARS-CoV-2 patients reported differed between children (< 5%) [8] and adults (5–17.9%) [9]. Large variations in prevalence were observed across countries and between age-groups among adults. Notably, data on asthma prevalence among children with SARS-CoV-2 is rare. Even with the relatively low sample size, our data supports findings of marginally lower SARS-CoV-2 infection rates among asthmatics compared to the general population [10]. These results overall may suggest that asthmatics are not at increased risk of COVID-19. However, a sizeable proportion of adult patients (20%) reported worsening of disease which was strongly associated with symptoms of depression (p = 0.002)—but unrelated to limited access to health care.

Other authors reported the risk of health deterioration especially in patients with chronic diseases due to missed or postponed appointments during the current pandemic [11]. Our data support the urgent need to investigate alternative care options for patients with chronic diseases, especially during the pandemic era. The World Health Organization states that wearing a mask of breathable material is safe, and the protective effect for the wearer is supposed to be a result of the reduced risk of virus load in case of exposure to an infectious individual [12]. However, wearing a facemask resulted in a feeling of difficult breathing across all age ranges in one-third to two-thirds of asthma patients in this survey. We did not examine the extent of discomfort in healthy control subjects, thus, disease-related differences cannot be asserted. We, therefore, cannot assess the additional asthma-specific contribution to breathing difficulties associated with the facemask. Since the type of mask (self-made, surgical, FFP) was unrelated to the reported subjective symptoms, these may be rather general discomfort than lung function impairment. Since the use of FFP2 masks has increased significantly in the population, future data will provide more differentiated insight into the impact on subjective and objective outcomes in asthma patients.

An intriguing observation is the high percentage of preschool-aged children with reported improvement in symptoms (32%, Fig. 1d). No worsening of symptoms was reported for any child. In this age-group, viral infections, mostly with rhinovirus, are the predominant trigger of the disease. While some improvements in preschool wheezers are seen in the summer months, the extent of change in this survey seems astonishing. Social distancing, the closing of daycare and kindergarden during lockdown have certainly contributed to a significant reduction in exposure to viral infections. However, since we do not have a direct comparison with data before the lockdown, we cannot quantify the impact of reduced exposure. In essence, the COVID-19 pandemic can be considered a natural experiment—allowing the assessment of the relative contribution of viral infections to asthma and pre-school morbidity at a young age.

### Conclusion

In our study, we show for the first time that the corona pandemic affects asthmatics of all age-groups differently. While we observed a high rate of problems caused by facemasks and a significant reduction in the use of routine medical care regardless of the age of our asthma patient, the impact on psychological health and asthma control was completely contrary. The corona related side effects on adults in terms of depression and disease progression is worrying. However, children hardly seem to suffer from the measures and sometimes even experience an improvement regarding their asthma control. This improvement might be contributed to a significant reduction in exposure to viral infections. Long-term follow-up may provide us with a better understanding of the influence of viral infections on the development of asthma in early childhood.

## Data Availability

The datasets used and analysed during the current study are available from the corresponding author on reasonable request.

## Abbreviations

COVID-19: Corona Virus Disease 2019
FFP2: Filtering face piece, type 2
PHQ-9: Patient Health Questionnaire 9
SARS-CoV-2: Severe acute respiratory syndrome-related coronavirus 2
